# ﻿Terrestrial crustaceans (Arthropoda, Crustacea): taxonomic diversity, terrestrial adaptations, and ecological functions

**DOI:** 10.3897/zookeys.1169.97812

**Published:** 2023-07-13

**Authors:** Ivan N. Marin, Alexei V. Tiunov

**Affiliations:** 1 A.N. Severtsov Institute of Ecology and Evolution, Russian Academy of Sciences, Moscow 119071, Russia A.N. Severtsov Institute of Ecology and Evolution, Russian Academy of Sciences Moscow Russia

**Keywords:** Crustacea, ecosystem engineers, food webs, morphological adaptations, soil animals, terrestrialization, trophic ecology

## Abstract

Terrestrial crustaceans are represented by approximately 4,900 species from six main lineages. The diversity of terrestrial taxa ranges from a few genera in Cladocera and Ostracoda to about a third of the known species in Isopoda. Crustaceans are among the smallest as well as the largest terrestrial arthropods. Tiny microcrustaceans (Branchiopoda, Ostracoda, Copepoda) are always associated with water films, while adult stages of macrocrustaceans (Isopoda, Amphipoda, Decapoda) spend most of their lives in terrestrial habitats, being independent of liquid water. Various adaptations in morphology, physiology, reproduction, and behavior allow them to thrive in virtually all geographic areas, including extremely arid habitats. The most derived terrestrial crustaceans have acquired highly developed visual and olfactory systems. The density of soil copepods is sometimes comparable to that of mites and springtails, while the total biomass of decapods on tropical islands can exceed that of mammals in tropical rainforests. During migrations, land crabs create record-breaking aggregations and biomass flows for terrestrial invertebrates. The ecological role of terrestrial microcrustaceans remains poorly studied, while omnivorous macrocrustaceans are important litter transformers and soil bioturbators, occasionally occupying the position of the top predators. Notably, crustaceans are the only group among terrestrial saprotrophic animals widely used by humans as food. Despite the great diversity and ecological impact, terrestrial crustaceans, except for woodlice, are often neglected by terrestrial ecologists. This review aims to narrow this gap discussing the diversity, abundance, adaptations to terrestrial lifestyle, trophic relationships and ecological functions, as well as the main methods used for sampling terrestrial crustaceans.

## ﻿Introduction

The subphylum Crustacea represents one of the largest and morphologically diverse taxa within the phylum Arthropoda (clade Mandibulata) with more than 70,000 known species, inhabiting all major ecosystems on Earth, except the airspace (Schram 1986). Some recent molecular studies show that the subphylum Crustacea is paraphyletic and includes all animals in the Pancrustacea clade, except for Hexapoda (Rota-Stabelli et al. 2010). However, some crustaceans of the Vericrustacea group (e.g., Anostraca, Copepoda, Malacostraca, and others) are closely related to insects (Insecta) than to other crustaceans from the Oligostraca clade (e.g., Ostracoda) (Koenemann et al. 2010; Regier et al. 2010). By other analysis, the clade Pancrustacea includes Hexapoda, being the most diverse group of animals on Earth (Bernot et al. 2022).

The main lineages of Crustacea appeared in the Cambrian time, being presumably marine aquatic animals (Chen et al. 2001; Schwentner et al. 2017). Since then, various lineages of crustaceans have tried to conquer the land, and many have succeeded. The first terrestrial crustaceans may have colonized the soil through marine basins during the Carboniferous period, ~ 360–300 Mya (Golonka and Ford 2000), and then filled ecological niches in moist and shaded soils in forests on coastal flood plains (Williams et al. 2006; Bennett 2008; Broly et al. 2013a). Their land colonization process occurred later than in the insects’ ancestors, which probably left the sea in the Lower Devonian, ~ 405 Mya (Grimaldi 2010; Watson-Zink 2021). The recent phylogenetic conclusions on Talitroidea have shown that their ancestors appeared no later than the Jurassic period, with the radiation of the ancestor taxa well established by the beginning of the Cretaceous period, ~ 140–138 Mya, being semi-terrestrial amphipods living close to the sea in swamps and mangrove forests (Myers and Lowry 2020). The first attested occurrences of Oniscidea (woodlice), the only modern group of Crustacea almost entirely composed of terrestrial forms, are recorded from the Early Cretaceous (100.5–66 Mya), while the paleobiogeographic context of fossil specimens and current biological considerations support a pre-Pangaea origin of the Oniscidea, in the Late Paleozoic, 350–250 Mya, most likely during the Carboniferous period (Carefoot and Taylor 1995; Tabacaru and Danielopol 1996; Broly et al. 2013a). Decapod crustaceans colonized the terrestrial habitats significantly later. For example, true crabs (Brachyura) independently colonized terrestrial habitats several times and separated from related marine/estuarine or freshwater relatives during the Late Cretaceous Period (100.5–66 Mya) (Watson-Zink 2021; Wolfe et al. 2022). Land-dwelling lifestyle has become the main factor of their further diversification, and numerous semi-terrestrial and terrestrial lineages radiated in the Early Eocene, which possibly coincided with global warming during the Paleocene-Eocene Thermal Maximum (~ 55 Mya) (Tsang et al. 2022). Colonization of terrestrial habitats has probably occurred relatively recently in Ostracoda and Branchiopoda, with marine eurytopic species colonized terrestrial niches from brackish and fresh waters in coastal floodplains (Williams et al. 2006; Bennett 2008).

Present-day terrestrial crustaceans thrive in very diverse habitats and may even be the predominant life form in some land ecosystems (Hansson et al. 2011). They were among the first invertebrates discovered and described by scientists. The land crab *Cancer ruricola* (= *Gecarcinusruricola*) and woodlouse *Oniscusasellus* were described by Linnaeus (1758) in “Systema Naturae”. European sand- and landhoppers, for example, *Talitrusgrillus* (= *Speziorchestiagrillus*), were also described at the dawn of the diversity studies (Bosc 1802; Latreille 1802; Montagu 1808). The first terrestrial ostracod *Mesocyprispubescens* and harpacticoids from genera *Epactophanes* and *Parastenocaris* were discovered in the early 20^th^ century from mosses and epiphytes of the cloud forests of Africa (Daday 1910) and Indonesia (Menzel 1916, 1921, 1923, 1926), respectively, while Chappuis (1928a, 1982b, 1930) described several tiny harpacticoids from mosses of the Himalayas’ foothills. The first truly terrestrial brachiopodans (= cladocerans), *Bryospilusrepens* and *Bryospilusbifidus* (Chydoridae), were discovered in 1980 in epiphytic mosses and the litter of cloud and rain forests of Puerto Rico, Venezuela, and New Zealand (Frey 1980). Nevertheless, crustaceans, except for woodlice, are not usually regarded as a group characteristic of terrestrial habitats. Despite their abundance and obvious importance in many habitats of tropical and ex-tropical terrestrial ecosystems, their ecological role and impact remain underestimated and undeservedly overlooked by “terrestrial” soil zoologists and ecologists (Fiers and Ghenne 2000).

In the review, we provide an overview of published data on the diversity, abundance, and ecological role of crustaceans living in terrestrial environments, excluding subterranean habitats, phytothelmata and other small water reservoirs, peat bogs, and other habitats providing a constant aquatic environment. We consider terrestrial those crustaceans whose adult stages spend most of their lives in terrestrial habitats more or less isolated from water sources. We aimed to show that crustaceans are among the functionally important components of terrestrial biodiversity, although their impact on ecosystem processes often remains underestimated. This review includes three main parts: 1) diversity and abundance in terrestrial habitats; 2) most important morphological, physiological, and behavioral adaptations; 3) trophic connections and functional role in ecosystems. In addition, we provide a brief description of the main methods that could be useful for sampling terrestrial crustaceans.

## ﻿Diversity and abundance in terrestrial habitats

### ﻿Habitats and distribution

Even well-drained terrestrial habitats contain a large amount of water, mainly in the form of surface water films and capillary soil moisture (Ghilarov 1956; Robinson et al. 2008). Water-filled pores and microfilms are also abundant in moist organic epigean substrates such as leaf litter or moss cushions. The total volume of this “cryptic water” is almost equal to the combined volume of freshwater lakes and rivers (Hutchinson 1957; Bittelli 2011).

Dependence on cryptic water underlies a traditional division of terrestrial crustaceans into two ecological (not phylogenetic) groups: microcrustaceans and macrocrustaceans (Table 1) (Hurley 1968; Little 1983).

**Table 1.** Comparative ecological characteristics of micro- and macrocrustaceans.

	Microcrustacea	Macrocrustacea
Taxa	Branchiopoda, Ostracoda, Copepoda	Malacostraca (Amphipoda, Isopoda, Decapoda)
Size	Total body length < 1 mm; body mass < 1 mg	Total body length from 2–3 mm to 120 mm; body mass up to 4 kg
Diversity	~ 220 known terrestrial or semi-terrestrial species	~ 4500 known terrestrial or semi-terrestrial species
Water	Depend on water films during the whole life cycle	Isopoda and Amphipoda mostly independent, Decapoda need liquid water for breeding
Habitats	Moist organic substrates, such as humid leaf litter and moss cushions, wet soils of shorelines and marshes. Branchiopoda and Ostracoda are confined mainly to warm regions	Almost everywhere, including high altitudes, arid deserts, Arctic, and sub-Antarctic tundra; soil, litter, arboreal habitats. Most of Amphipoda and Decapoda are confined to warm regions

#### Microcrustaceans

Microcrustaceans, represented by Branchiopoda (Cladocera), Ostracoda, and Copepoda, are tiny arthropods with a total body length typically < 1 mm, which are dependent on and associated with surface water films or pore water. These microhabitats are often discrete and restricted in volume and therefore are strongly influenced by wetting, drying, precipitation, drainage, and evaporation. Branchiopods and ostracods are known for permanently wet habitats in tropical and subtropical cloud and rain forests, where they live in thin films and small accumulations of water on the vegetation and forest floor. The most important condition for the survival of these animals is seemingly regular precipitation (Harding 1953, 1955; Schornikov 1969; Frey 1980; Martens et al. 2004; Pinto et al. 2005a). Terrestrial Copepoda (Harpacticoida and Cyclopoida) have a wider distribution range. Harpacticoids have been found in habitats containing only a small amount of capillary water, while the ability to encyst allows them to survive persistent droughts, as well as to spread over long distances with the help of wind or with clumps of moss carried by other animals (Deevey 1941; Glime 2017a). In addition to moist tropical and temperate zones, where they are quite diverse (Dahms and Qian 2004; Martens et al. 2008), they are known for boreal and polar meadow/tundra soils and coastal environments, extending to Arctic and sub-Antarctic (Reid 1986; Flössner 1992; Hansson et al. 1996; Pugh et al. 2002; Marin and Palatov 2023). They have been found also in mountain habitats, for example, harpacticoid *Elaphoidellapseudocornuta* is known from the leaf litter of the wet forests of Nepal at an altitude of 1900–3900 m a.s.l. (Dumont and Maas 1988). Microcrustaceans are probably very widespread in terrestrial biotopes where there is at least a small amount of pore water and extremely low or high temperatures are not reached. Due to their small size, they rarely attract the attention of soil zoologists. Occasionally, the presence of microcrustaceans in soil and other terrestrial habitats is considered an artifact (Fiers 2013) and the actual distribution of microcrustaceans in terrestrial environments is probably underestimated (Fiers and Ghenne 2000).

#### Macrocrustaceans

Macrocrustaceans, represented by malacostracan orders Amphipoda, Isopoda, and Decapoda, are relatively large arthropods, usually (2–300 mm in the total body length), which are significantly less dependent on water films, pore water, and even soil moisture (Hurley 1968; Little 1983). These crustaceans densely populate coastal marine zone (Bliss and Mantel 1968; Friend and Richardson 1986; Myers and Lowry 2020), tropical and subtropical marine islands (Green 1997; Lindquist et al. 2009), and temperate inland forests, where they occur from the soil of the forest floor to the top tier of the canopy (Richardson 1992; Taylor et al. 1993; Biju Kumar et al. 2017; Ng and Ng 2018; Wongkamhaeng et al. 2018). In favorable terrestrial environments, crustaceans have undergone profound morphological and ecological speciation, and occupy various ecological niches (Schmalfuss 1984, 2003; Vilisics et al. 2007; Schmidt 2008; Hsu et al. 2018). Specific morphological adaptations and behavioral reactions of macrocrustaceans (see below) allowed them to colonize a great variety of arid land habitats (Edney 1968; Lindqvist 1968; Hornung 2011). Many species thrive in extreme abiotic conditions, such as high-altitude and cold Arctic or sub-Antarctic regions, dry and acidic habitats (Richardson and Jackson 1995; Serejo 2004, 2009; Greenslade et al. 2008; Lowry and Coleman 2012). The highest altitude dwelling crab, *Potamonautesloveni* from Kenya and Uganda lives in terrestrial habitats up to 3060 m a.s.l. (Cumberlidge and Clark 2010), while crabs *Geothelphusahaituan* are known from cloud forests growing at approximately 2000 m a.s.l. in mountains of Taiwan (Chen et al. 2007). Woodlice have been reported from altitudes higher than 4000 m a.s.l., reaching high abundance and density there (Sfenthourakis et al. 2008). The woodlouse *Protracheoniscusnivalis* Verhoeff, 1936 inhabits cloud forests at altitudes reaching 4725 m a.s.l. in Ladakh and an unidentified species was found in mountains of the northwest Himalayas, where oxygen can drop to 60% of the sea-level pressure (Beron 1997, 2008; Hegna and Lazo-Wasem 2010).

Nevertheless, the diversity of ecological niches and biotopes occupied by macrocrustaceans is largely determined by physical environmental factors, such as moisture and temperature. For example, the distribution of woodlice towards the north is limited by the duration of the warm period, and the highest diversity of woodlice on the territory of the former USSR was observed between isoclines of 180 and 210 days with temperature > 10 °C (Kuznetsova and Gongalsky 2012), while the upper limits of temperature tolerance estimated for landhoppers varied between 29.5 °C and 39.5 °C (Gaston and Spicer 1998; Ulian and Mendes 1988; Cowling et al. 2003, 2004). While most terrestrial crustaceans prefer warm conditions, some isopods and amphipods are cold-resistant (Tanaka and Udagawa 1993; Moore et al. 1995; Greenslade et al. 2008). For example, Arctic talitroids of the genus *Orchestia* can survive at temperatures below 0 °C, and even -8 °C (Moore and Francis 1986a, 1986b). The critical relative humidity for most talitroids, below which they show desiccation stress, is close to 95–100%, which makes them dependent on moist leaf litter and soil microhabitats (Lazo-Wasem 1984; Cowling et al. 2003). Synanthropic landhopper *Talitroidestopitotum* is considered one of the most tolerant to low humidity but can survive only for 50 h at a relative humidity of 87% (Ulian and Mendes 1988). In contrast, land crab Holthuisana (Austrothelphusa) transversa can survive in arid clay soils of the Australian desert (Greenaway and MacMillen 1978; MacMillen and Greenaway 1978; Waltham 2016), while woodlouse *Hemilepistusreaumurii* inhabits dry loess soils in the Sahara Desert and Negev Desert (Shachak et al. 1976; Dubinsky et al. 1979).

In addition to the forest floor, macrocrustaceans are found in various aboveground habitats, but for many groups such records are still casual. Although most woodlice live in soils and litter layers, some members of the Philosciidae, Armadillidae and Trachelipodidae are arboreal (Paoletti et al. 1991; Paoletti and Hassall 1999). In temperate forests woodlice *Philosciaaffinis*, *Philosciamuscorum*, and *Porcellioscaber* are frequently found in the forest canopy, on tree bark, leaves, and branches, not only when the forest floor is inundated or waterlogged (Favretto et al. 1988; Warburg 1993). Arboricolous woodlouse *Atracheodillomarmorivagus* lives on *Carapagrandiflora* in Congo and Rwanda (Schmidt 1999), while South African *Alloniscusmarinus* also lives and feeds on green leaves of the bietou bush Chrysanthemoides (Osteospermum) monilifera (Glazier and Kleynhans 2015). *Pseudolaureolaatlantica*, endemic woodlouse to St Helena Island, requires the closed canopy and high humidity conditions of black cabbage tree woodland (*Melanodendronintegrifolium*), living an arboreal lifestyle on the fern understorey (Dutton 2017a, b). Arboreal forms are known for talitroids; for example, *Hawaiorchestiagagnei* and *Platorchestiapickeringi* were found in leaf axils of *Freycinetiaarborea* well above ground (Richardson 1992). *Allorchestoidesrosea* lives among leaves and fibers of the estuarine *Nypa* palm (*Nypafruticans*) in Thailand (Wongkamhaeng et al. 2018). A truly high diversity of tree-dwelling forms is reached in crabs (Decapoda: Brachyura). Some true crabs, such as Gecarcinucidae (long-legged tree crabs), Potamonautidae, Parathelphusidae, Pseudothelphusidae, and Sesarmidae (so-called “vampire crabs”) climb into the crowns of trees but must descend into water reservoirs for reproduction (Schubart et al. 2003, 2009; Cumberlidge et al. 2005; Ng et al. 2015a, 2015b; Wehrtmann et al. 2016; Ng 2017). Some long-legged Asian crabs, e.g., *Calcipotamon*, *Tiwaripotamon*, and *Neotiwaripotamon*, inhabit karstic mountains and massifs where they hide in the water-filled crevices of limestone outcrops, going out at night for feeding in the forest floor (Shih and Do 2014; Do et al. 2016; Huang et al. 2020). Malaysian crabs *Arachnothelphusamerarapensis* and *Arachnothelphusaterrapes* (Ng 1991; Grinang et al. 2015; Ng and Ng 2018) and *Kanimaranjandu*, inhabiting holes inside large *Terminalia paniculata* trees in the Western Ghats (Biju Kumar et al. 2017), are fully arboreal, and can even breed in the small water-filled reservoirs in tree hollows (Ng 1995; Ng and Liu 2003). Jamaican *Metopauliasdepressus*, one of the most advanced arboreal crabs, is showing features of eusocial behavior, and protect their plants and larvae (Diesel 1989; Diesel and Schuh 1993; Diesel and Schubart 2000, 2007).

Many terrestrial macrocrustaceans are invaders or synanthropes that successfully colonize transformed ecosystems, urban areas, and other anthropogenic habitats such as parks and gardens (Perger et al. 2013; Perger 2014). Woodlice are among the most numerous groups of epigeic arthropods in the transformed habitats (Philpott et al. 2014; Hornung et al. 2015), where the likelihood of successful settlement of invasive species is increased due to suppressed activity of native predators or competitors (Sorensen and Burkett 1977; Szlavecz et al. 2018). From 20% to 90% of species living in transformed habitats of city parks in Japan are represented by terrestrial isopods, mostly invasive species (Lee and Kwon 2015; Giurginca et al. 2017). The most famous invasive landhopper species, *Talitroidestopitotum*, formerly endemic to the Indo-Pacific region, is now distributed worldwide through the marketing of exotic plants (Álvarez et al. 2000; Eutrópio and Krohling 2013; Arias-Pineda and Tristancho 2017). Better resistance to drying and the ability to detect and occupy wet shelters in drier habitats allow *Talitroidestopitotum* to displace native talitroid species (Friend and Lam 1985; Richardson 1992). Among the invasive talitroids are also *Platorchestiaplatensis* (Serejo and Lowry 2008; Simpson 2011; Hupało and Grabowski 2018), *Cryptorchestiacavimana* (Konopacka et al. 2009), *Brevitalitrushortulanus*, *Talitroidesalluaudi* (Lincoln 1979; Jazdzewski et al. 2004), and *Arcitalitrus* spp. (Richardson 1980). On the other hand, local communities of native terrestrial crustaceans, especially on isolated oceanic islands, are often affected by various invasive terrestrial invertebrates, such as the predatory nemertean *Geonemertespelaensis* (Shinobe et al. 2017), the land snail *Lissachatinafulica* (Lake and O’Dowd 1991), and the yellow crazy ant, *Anoplolepisgracilipes* (O’Dowd et al. 2003).

### ﻿Diversity

**Figure 1.**
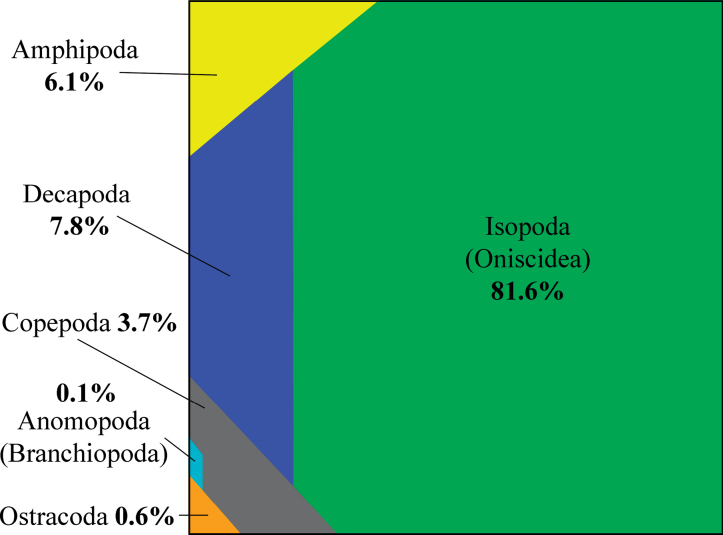
Proportions (%) of all known species of terrestrial Crustacea among different lineages (orders).

The proportion of terrestrial or semi-terrestrial species among different orders of Crustacea varies greatly, and in some lineages might be underestimated. The diversity of terrestrial Branchiopoda (Sousa et al. 2017) and Ostracoda (Pinto et al. 2004, 2005a, 2005b, 2008; Karanovic et al. 2012) is low, represented by only a few species or genera. Approximately 100 terrestrial or semi-terrestrial species have been described in Copepoda, mainly Harpacticoida (Reid 1986, 2001) (Table 2). Overall, terrestrial forms represent < 0.1% of the total diversity of these mainly aquatic taxa. Among macrocrustaceans, ~ 1.7% of the total diversity of order Decapoda (Little 1983; Greenaway 2003), ~ 3% of the order Amphipoda (Hurley 1968; Friend and Richardson 1986; Myers and Lowry 2020), and up to 33% of the order Isopoda (Taiti 2004; Poore and Bruce 2012; Sfenthourakis and Taiti 2015) are presently known as terrestrial species (Table 3). Among the total diversity of terrestrial crustaceans (~ 4,900 species), Branchiopoda and Ostracoda account for 0.1% and 0.6% of all species, respectively. Copepoda, Amphipoda, and Decapoda account for approximately 3.7%, 6.1%, and 7.8%, respectively, while the main diversity (81.6%) belongs to woodlice (Isopoda) (Fig. 1).

**Table 2.** Taxa with terrestrial or semi-terrestrial forms among microcrustaceans (Branchiopoda, Ostracoda, Copepoda).

Families	Genera
**Branchiopoda (Cladocera), order Anomopoda (3 genera)**	
Chydoridae	*Bryospilus*, *Nicsmirnovius*, *Monospilus*
**Ostracoda, order Podocopida (12 genera)**	
Cyprididae	*Austromesocypris*, *Bryocypris*, *Callistocypris*, *Mesocypris*, *Scottia*
Candonidae	*Caaporacandona*, *Terrestricandona*, *Terrestricypris*
Limnocytheridae	*Intrepidocythere*
Terrestricytheridae	*Terrestricythere*
Darwinulidae	*Penthesilenula*, *Vestalenula*
**Copepoda, order Harpacticoida (18 genera)**	
Parastenocarididae	*Remaneicaris*, *Forficatocaris*, *Murunducaris*
Canthocamptidae	*Canthocamptus*, *Bryocamptus*, *Epactophanes*, *Fibulacamptus*, *Maraenobiotus*, *Moraria*, *Gulcamptus*, *Remaneicaris*, *Pindamoraria*, *Eirinicaris*, *Elaphoidella*, Attheyella (Chappuisiella), *Antarctobiotus*
Phyllognathopodidae	*Phyllognathopus*, *Parbatocamptus*
**Copepoda, order Cyclopoida (10 genera)**	
Cyclopidae	*Bryocyclops*, *Virbiocyclops*, *Paracyclops*, *Goniocyclops*, *Graeteriella*, *Ectocyclops*, *Menzeliella*, *Metacyclops*, *Muscocyclops*, *Olmeccyclops*

**Table 3.** Taxa with terrestrial or semi-terrestrial forms among macrocrustaceans (Malacostraca).

Superfamilies or families	Families or genera
**Order Amphipoda (125+ genera)**	
Superfamily Talitroidea	Arcitalitroidae (15 genera), Protorchestiidae (6 genera), Uhlorchestiidae (1 genus), Brevitalitroidae (8 genera), Curiotalitroidae (1 genus), Makawidae (22 genera), Talitroidae (72 genera)
**Order Isopoda (554+ genera)**	
Suborder Oniscidea	Agnaridae (14 genera), Alloniscidae (1 genus), Armadillidae (81 genera), Armadillidiidae (16 genera), Balloniscidae (2 genera), Bathytropidae (7 genera), Berytoniscidae (1 genus), Bisilvestriidae (1 genus), Cylisticidae (4 genera), Delatorreiidae (3 genera), Detonidae (3 genera), Dubioniscidae (3 genera), Eubelidae (50 genera), Halophilosciidae (3 genera), Hekelidae (1 genus), Irmaosidae (1 genus), Ligiidae (6 genera), Mesoniscidae (1 genus), Olibrinidae (5 genera), Oniscidae (6 genera), Oniscidea incertae sedis (30 genera), Paraplatyarthridae (1 genus), Philosciidae (115 genera), Platyarthridae (8 genera), Porcellionidae (19 genera), Pudeoniscidae (4 genera), Rhyscotidae (2 genera) Schoebliidae (1 genus), Scleropactidae (28 genera), Scyphacidae (6 genera), Spelaeoniscidae (9 genera), Stenoniscidae (3 genera), Styloniscidae (17 genera), Tendosphaeridae (3 genera), Titanid ae (5 genera), Trachelipodidae (8 genera), Trichoniscidae (87 genera), Turanoniscidae (1 genus), Tylidae (2 genera)
Suborder Phreatoicidea	Phreatoicidae (1-2 terrestrial genera)
**Order Decapoda**	
(most mangrove mud-dwelling and costal crabs are excluded)	
**Infraorder Astacidea (5 genera)**	
Cambaridae	*Distocambarus* (= *Fitzcambarus*)
Parastacidae	*Engaeus*, *Euastacus*, *Parastacus*, *Virilastacus*
**Infraorder Caridea (1 genus)**	
Merguiidae	*Merguia*
**Infraorder Anomura (2 genera)**	
Coenobitidae	*Birgus*, *Coenobita*
**Infraorder Brachyura (73+ genera)**	
Gecarcinidae	*Cardisoma*, *Discoplax*, *Gecarcinus*, *Gecarcoidea*, *Johngarthia*, *Tuerkayana*
Gecarcinucidae	*Arachnothelphusa*, *Ceylonthelphusa*, Holthuisana (Austrothelphusa), *Sayamia*, *Sundathelphusa*, *Terrathelphusa*, *Thelphusula*
Grapsidae	*Geograpsus*, *Goniopsis*, *Metopograpsus*
Parathelphusidae	*Esanthelphusa*, *Oziotelphusa*, *Parathelphusa*, *Perbrinckia*
Potamidae	*Binhthuanomon*, *Calcipotamon*, *Candidiopotamon*, *Carpomon*, *Chinapotamon*, *Dromothelphusa*, *Gempala*, *Geothelphusa*, *Indochinamon*, *Johora*, *Krishnamon*, *Nanhaipotamon*, *Neotiwaripotamon*, *Phaibulamon*, *Pudaengon*, *Qianguimon*, *Rathbunamon*, *Ryukyum*, *Socotra*, *Somanniathelphusa*, *Thaiphusa*, *Thaipotamon*, *Tiwaripotamon*
Potamonautidae	*Globonautes*, *Liberonautes*, *Madagapotamon*, *Malagasya*, *Potamonautes*, *Sudanonautes*
Pseudothelphusidae	*Epilobocera*, *Guinotia*, *Ptychophallus*
Sesarmidae	*Aratus*, *Armases*, *Chiromantes*, *Episesarma*, *Geosesarma*, *Geosesarmamirum*, *Haberma*, *Karstama*, *Labuanium*, *Metasesarma*, *Metopaulias*, *Neosarmatium*, *Parasesarma*, *Perisesarma*, *Scandarma*, *Sesarmoides*, *Sesarmops*, *Selatium*, *Tiomanium*
Trichodactylidae	*Trichodactylus*
Varunidae	*Chasmagnathus*

Branchiopoda (order Anomopoda) appear to be the least diverse and adapted forms among terrestrial crustaceans and retain a mostly ancestral aquatic lifestyle. Currently, only five species from three genera are reported as semi-terrestrial (Table 2), living in wet mosses growing in primary cloud/rain forests of New Zealand, Cameroon, Puerto Rico, and Venezuela, and wet soils of Cerrado biotopes in Brazil (Frey 1980; Cammaerts and Mertens 1999; Chiambeng and Dumont 1999; Sousa et al. 2017).

The list of terrestrial Ostracoda presently consists of > 30 species from 12 genera, known from leaf litter and wet mosses of tropical and subtropical forests, the spray zone of waterfalls (Martens et al. 2004; Pinto et al. 2004, 2005a, 2005b, 2008; Karanovic et al. 2012), and some coastal habitats, such as coastal wood and algae deposits in Kuril Islands (Schornikov 1969). These genera represent several unrelated lineages adapted to the terrestrial lifestyle, but their phylogeny and zoogeography are still poorly understood.

The known diversity of terrestrial and semi-terrestrial Copepoda includes 18 harpacticoid and 10 cyclopoid genera found in soil, leaf litter, and other moist habitats in tropical and temperate zones worldwide (Reid 1986, 1993, 2001; Dumont and Mass 1988; Corgosinho et al. 2017). Records of these animals in terrestrial habitats are rare and irregular, although thorough studies of soil biota often revealed a greater diversity than could be expected (Fiers and Ghenne 2000; Fiers and Jocque 2013).

The diversity of terrestrial Amphipoda, or sand- and landhoppers (Talitroidea) includes ~ 300 described species from seven families with at least 125 genera (Table 3) inhabiting various terrestrial, intertidal, and supralittoral habitats from sandy beaches to leaf litter of lowland and highland forests worldwide (Serejo and Lowry 2008; Lowry and Myers 2019; Myers and Lowry 2020).

Isopoda are the most advanced, adapted, and successful land colonizers presented by the cosmopolitan suborder Oniscidea (woodlice). Oniscidea include ~ 40 families with ~ 552 genera and ~ 4,000 described species, while an estimated diversity is close to 5,000–7,000 extant species (Schmalfuss 2003; Javidkar et al. 2015; Sfenthourakis and Taiti 2015). Due to their wide distribution, diversity, and high abundance, woodlice are the best-studied terrestrial crustaceans. In addition, there are several semi-terrestrial burrowing species of the genus *Phreatoicopsis* (Phreatoicidea: Amphisopodidae) known from wetlands or swamps of the Grampians National Park, Australia (Spencer and Hall 1896; Wilson and Keable 2002).

Terrestrial and semi-terrestrial Decapoda are represented by four infraorders: Reptantia (crayfish) (~ 60 species), Anomura (hermit crabs) (~ 18 species), Caridea (shrimp) (2–4 species), and Brachyura (crabs) (~ 300 species) (Table 3). Terrestrial representatives of Reptantia are limited to some members of the Cambaridae and Parastacidae, living in damp soils and nearly completely independent of surface waters. The evolution of terrestriality is especially characteristic of the genera *Engaeus* (> 35 species), some *Euastacus* and *Cherax* species from Australia and Tasmania, *Parastacus* (> 14 species) and *Virilastacus* (4 species) from South America (mainly Chile), and *Distocambarus* (= *Fitzcambarus*) (4 species) from the southeastern USA (Georgia/North Carolina) (Horwitz 1990; Furse and Wild 2002; Rudolph and Crandall 2012; Reynolds et al. 2013; McCormack and Raadik 2021). Most of these crayfish build deep and complex burrows that are not associated with permanent water reservoirs (Welch and Eversole 2006). They spend their entire life cycle inside the burrows feeding on roots and leaves, collecting the latter from the soil surface, and making frequent excursions from the burrows at night or after rain floods or snowmelts, as do Fallicambarus (Creaserinus) fodiens (Decapoda: Cambaridae) in the USA and Canada (Suter and Richardson 1977; Growns and Richardson 1988; Richardson and Swain 1990; Norrocky 1991; Guiaşu 2007; Graham et al. 2022). Infraorder Anomura is represented in terrestrial habitats by hermit crabs of the family Coenobitidae, including the genus *Coenobita* (> 17 species) and the coconut crab *Birguslatro* (Rahayu et al. 2016). Among Caridea, two shrimp species of the genus *Merguia* (Merguiidae) show a semi-terrestrial lifestyle, inhabiting fringe or riverine mangrove swamps and climbing tree trunks at night during the low tides (Abele 1970; Vannini and Oluoch 1993). In addition, two species of the genus *Potamalpheops*, the Asian *P.kisi*, and the Australian *P.hanleyi*, were also found in semi-terrestrial habitats of mangrove forests (Bruce 1991; Marin 2021). The highest grades of adaptations to terrestrial lifestyle within the Brachyura are realized in Gecarcinidae (> 20 species), Sesarmidae (> 250 species), and some representatives of Gecarcinucidae, Potamidae, Gecarcinidae, Potamonautidae, Pseudothelphusidae, and Trichodactylidae (Bright and Hogue 1972; Biju Kumar et al. 2017; Ng and Ng 2018); although the term “land crab” is often used to mean solely the representatives of the family Gecarcinidae (Burggren and McMahon 1988; De Grave et al. 2009).

We would like to devote a separate paragraph to peculiar records of crustaceans in soil communities, which seem unusual even in the light of this review. In addition to Talitroidea, several amphipod species of genera *Niphargus* and *Microniphargus* (Niphargidae) have been reported from soil habitats (Lagidze et al. 1974; Turquin 1983; Hudec et al. 2017). *Niphargustalikadzei* was described as “the first true soil-dwelling *Niphargus* species” (Lagidze et al. 1974) from Georgia (Caucasus), an extremely aberrant species *Niphargusrhenorhodanensis* from interstitial and soil capillary cavities in France (Mathieu et al. 1994), while several *Microniphargus* species have been occasionally recorded in soil samples in Ireland (Arnscheidt et al. 2008, 2012). The Amphipod *Rudolphiamacrodactylus* (Paraleptamphopidae) was described from the soil burrows of semi-terrestrial crayfish *Virilastacusrucapihuelensis* (Parastacidae) and surrounding peat bogs in Chile (Grosso and Peralta 2009). A tiny amphipod of the genus *Palearcticarellus* (Crangonyctidae) has also been recorded from a wet moss around springs in the Kurai highland valley (steppe) in Altai, Russia, but not in the spring itself, where they were likely preyed upon by larger species of the genus *Gammarus* (Palatov and Marin 2020). Even more strange is the record of Branchiopoda in soil samples (Battigelli et al. 1994). All these animals are characteristic of small epigean (Brachiopoda) or subterranean (Niphargidae) water reservoirs (e.g., Fišer 2019), and probably should not be considered as true soil inhabitants, although our knowledge of the biology of these crustaceans is still strongly limited.

### ﻿Abundance (density and biomass)

Terrestrial crustaceans can reach high densities and abundance (Table 4), although for some groups, namely Brachiopoda (Anomopoda) and Ostracoda, such data are not yet available. Small-sized Copepoda, mainly harpacticoids, were reported to be “surprisingly abundant” in many terrestrial and semi-terrestrial habitats (Plowman 2006; Fiers and Ghenne 2000; Reid 2001; Reid and Rocha 2003), reaching up to 3% of all sampled animals (Battigelli et al. 1992; Fiers 2013). The density of Copepoda (54,400–93,600 ind/m^2^) was comparable to that of most abundant soil microarthropods, i.e., Acari (38,600–189,000 ind/m^2^) and Collembola (34,800–140,300 ind/m^2^) in the wet cedar-hemlock forest in British Columbia (Battigelli et al. 1992). The biomass of these animals has been rarely measured. Schaefer and Schauermann (1990) reported a dry mass of harpacticoids of ~ 0.6–2.0 mg/m^2^ in two beech forests in Germany with a density of 3,900–3,300 ind/m^2^. Based on recorded densities, the biomass of microcrustaceans can be much higher in some habitats.

**Table 4.** The maximum known abundance (density or biomass) of terrestrial crustaceans.

Species or taxa	Habitat	Abundance	Reference
**Microcrustacea**			
Harpacticoid copepods, probably one species	Beach forests in Germany	3,300–3,900 ind/m^2^ 0.6–2.0 mg d.wt./m^2^	Schaeffer and Schauermann 1990
Copepoda	Conifer subalpine forest in France	3,700 ind/m^2^	Bernier and Gillet 2012
Different harpacticoids	Canadian tundra	>6,500 ind/m^2^	Bliss et al. 1973
Harpacticoid *Forficatocarisschadeni*	Wet campo marshes of central Brazil	>178,000 ind/m^2^	Reid 1982
Copepoda, mainly harpacticoids	Wet cedar-hemlock forest in British Columbia	>54,400–93,600 ind/m^2^	Battigelli et al. 1992
**Macrocrustacea**			
**Landhoppers (Amphipoda: Talitroidea)**			
*Makawehurleyi*	New Zealand forests	1230–2670 ind/m^2^	Duncan 1994
Several syntopic talitroids	Tasmanian forests	>10,000 ind/m^2^	Friend and Richardson 1987
*Allorchestescompressa*	The coast of Western Australia	110 ind/g of algae remnants	Lenanton et al. 1982
*Bellorchestiaquoyana* (= *Talorchestiaquoyana*)	Coastal sand beaches in New Zealand	11.8 g/m^2^	Marsden 1991
**Woodlice (Isopoda: Oniscidea)**			
Woodlice	Temperate forests	35–630 ind/m^2^	Topp et al. 2006
*Atlantosciafloridana*	Semi-deciduous forest of Southern Brazil	1040 ind/m^2^	Araujo and Bond-Buckup 2005
Woodlice	Calcareous grasslands	800–3000 ind/m^2^	Paoletti and Hassall 1999; Gongalsky et al. 2005
*Porcellioscaber*	Northern France	5070 ind/m^2^ in aggregations	Broly et al. 2016
Desert woodlice *Hemilepistusreaumurii*	Deserts of Northern Africa	50 ind/m^2^ and 2 g/m^2^ in aggregations	Markwiese et al. 2000
Syntopic *Atlantosciafloridana* + *Balloniscusglaber*	Semi-deciduous forest of Southern Brazil	2.56 g/m^2^	Quadros and Araujo 2008
Woodlice	Temperate forests	0.09–0.35 g/m^2^	White 1968
**Crabs and hermit crabs (Decapoda)**			
*Gecarcinusquadratus*	Mainland forests of Costa-Rica	0.8–6 ind/m^2^	Sherman 2002, 2003; Lindquist and Carroll 2004; Lindquist et al. 2009
*Birguslatro*	Christmas Island	41–166 ind/ha	Rumpff 1986; Schiller 1988
*Gecarcinuslateralis*	Tropical semi-deciduous forests of Central America and Florida	1–3 ind/m^2^ and 2 burrows/m^2^	Bliss et al. 1978; Britton et al. 1982; Delfosse 1990; Kellman and Delfosse 1993
*Gecarcoideanatalis*	Christmas Island	1.3–2.6 ind/m^2^ (migrations) and 1.8 burrows/m^2^, with the estimated peak biomass close to 113.7–145.4 g/m^2^	Hicks 1985; O’Dowd and Lake 1991; Green et al. 1997; Adamczewska and Morris 2001
*Cardisomacrassum*	Mexico	the average density 1.66 burrow/m^2^	Vázquez-López et al. 2014
*Cardisomaguanhumi*	Venezuelan coastline and Florida	5.48 burrows/m^2^ (Venezuella) and ≥ 200 g/m^2^ (Florida)	Gifford 1962; Green 1997; Carmona-Suárez 2011
*Gecarcinusplanatus*	Clipperton Atoll	up to 6 ind/m^2^	Ehrhardt and Niaussat 1970; Turkay 1973; Wolcott 1988
*Coenobitarugosus*	Andaman Coast of Thailand	up to 8.4 ind/m^2^	Bundhitwongrut et al. 2014
*Coenobitaclypeatus*	Bahamian islands	~ 14.3 crabs/m^2^	Morrison and Spiller 2006
Sympatric *Coenobita* spp.	Vegetated area of Bahamian islands	46 ind/m^2^ in dense agglomerations	Morrison and Spiller 2006
*Ucaannulipes*	Coastal habitats of East Africa	175 ind/m^2^	Skov et al. 2002

Terrestrial Amphipoda and Isopoda are medium-sized arthropods (typically ≤ 30 mm in body length and dry weight not exceeding 50–70 mg). Their abundance and biomass can be comparable to those of millipedes and other co-dimensional representatives of soil macrofauna (David et al. 1999; Messelink and Bloemhard 2007; David and Handa 2010) (Table 4). The highest known biomass is reported for the desert woodlouse *Hemilepistusreaumurii* (~ 20 kg/ha with a population density of ~ 50 ind/m^2^), which is comparable to the combined biomass of desert mammals (~ 40 kg/ha) in the same habitats (Markwiese et al. 2001). This impressive statistic can be somewhat overestimated as it reflects densities of woodlice aggregations observed at the microhabitat scale (Broly et al. 2012).

Terrestrial Decapoda also achieve significant abundance, as well as the highest values of biomass among terrestrial arthropods, also being the heaviest of all terrestrial arthropods. In particular, the robber crab *Birguslatro* is the largest terrestrial arthropod, reaching 1 m in length from leg to leg and ~ 12 cm in width of the carapace with a weight of up to 4.0 kg (Brown and Fielder 1991; Drew et al. 2010, 2013). With a relatively large size (2–12 cm of carapace width and weight occasionally > 500 g) and a density often exceeding 1 ind/m^2^, the total biomass of land crabs can reach 1000 kg/ha and higher, especially during the annual breeding migrations (see below). In tropical island and inland forests, the biomass of terrestrial decapods released in the absence of natural enemies and competitors (Green 1997; Lindquist et al. 2009), can exceed the total biomass of animals reported in tropical rain forests in Costa Rica (115 kg/ha; Odum et al. 1970) and the central Amazon (210 kg/ha; Fittkau and Klinge 1973) (Table 4).

Summarizing, both micro- and macrocrustaceans are widely distributed in terrestrial environments, with the greatest diversity and abundance in warm and humid habitats, such as tropical and subtropical coastal forests. Large Decapoda species reach the maximum density and biomass on well-isolated tropical islands, which should be likely ascribed to the absence of competitors and predators, like mammals and forest birds. Medium-sized landhoppers and especially woodlice are acknowledged components of soil macrofauna in temperate and even subarctic ecosystems. Tiny harpacticoid and cyclopoid copepods are common members of the soil mesofauna and possibly occupy more ecological niches than is usually assumed, but their density and biomass are still underestimated.

#### Additional literature on the topic

Diversity, distribution, and evolution of woodlice are reviewed in Edney (1954), Warburg (1993), Sfenthourakis et al. (2004, 2020), Loureiro et al. (2005), and Hornung (2011); talitroids in Richardson and Swain (2000), terrestrial crabs in Burggren and McMahon (1988), crayfish in Reynolds et al (2013), and anomurans in Greenaway (2003).

### ﻿Adaptations to a terrestrial lifestyle

The transition from aquatic habitats to a terrestrial lifestyle required numerous adaptations in morphology, respiratory physiology, osmoregulation and water balance, excretion, respiration, sensory perception, thermoregulation, molting, reproduction, and behavior. Terrestrial crustaceans are in general morphologically similar to their aquatic ancestors, with morphological pre-adaptations to the terrestrial lifestyle differing in the main lineages. These adaptations can be categorized into five classes ranging from T1 (lowest) to T5 (highest) depending on the degree of independence from immersion in water and the need for access to water for breeding (Powers and Bliss 1983), although this classification is currently rarely used. Schubart et al. (2000) proposed an alternative system that includes three simplified degrees of terrestriality as follows: (A) terrestrial adults with marine larvae, (B) limnic-terrestrial adults (spend most of their lives in or near freshwater) with marine larvae, and (C) adults that breed in inland waters and hence are independent from the ocean. This classification is based on the paths of land penetration, which are reflected in the modern biology and ecology of crustaceans. Taxa derived from the marine environment must release larvae into the sea, returning to the mainland as the last zoea/megalopa stages, while taxa of freshwater origin mostly have reduced or abbreviated development and can live in terrestrial habitats far from the sea coastline (Wolcott 1988).

Studies on the mechanisms of terrestrialization rarely concern microcrustaceans, which have a very limited set of specific adaptations (e.g., Cammaerts and Mertens 1999; Chiambeng and Dumont 1999). Therefore, most examples below are representatives of macrocrustaceans.

### ﻿Morphological adaptations

#### Air-breathing structures and cuticle

Adaptations for air-breathing and preventing evaporation, a prerequisite for the terrestrial lifestyle, are represented by pleopodal lungs, or “pseudotrachea”, in woodlice, ancestrally derived from pleopodal gills, which can be conditionally categorized into three types: dorsal respiratory fields, uncovered, and covered lungs (Schmidt and Wägele 2001; Paoli et al. 2002; Hornung 2011; Csonka et al. 2013; Ernst et al. 2020; Sfenthourakis et al. 2020). Similar structures are presented on the abdomen and posterior surface of the carapace in terrestrial hermit crabs (Morris 2002; Farrelly and Greenaway 2005). Because the water content is related to the body mass, and water loss is proportional to the body surface, the loss of water through pleopods is most critical for small-size species, while larger species lose relatively less water through their pleopods and cuticle. Passive respiration using pleopods (in contrast to abdominal/branchiostegal lungs in Decapoda or tracheae in Insecta; Schmidt and Wägele 2001; Garwood and Edgecombe 2011) and the need to enforce the thin cuticle ensured further ecological diversification in woodlice. A trend of gill reduction is also described in talitroids (Moore and Taylor 1984; Richardson 1998). Despite elaborate adaptations, land-dwelling woodlice and talitroids are still feebly protected from desiccation, primarily due to the absence of waxy cuticle (Hadley and Quinlan 1984). The presence of specific epicuticular lipids reduces water loss due to evaporation in woodlice of the genera *Buddelundia* (arid regions of Australia), *Hemilepistus* (Sahara and Negev deserts), and especially in *Venezilloarizonicus* (Arizona desert) (Cloudsley-Thompson 1956, 1975, 1988; Warburg 1965a, 1965b). Furthermore, some woodlice possess air-breathing organs remarkably similar to tracheae in insects that expand and enter the thoracic body trunk (see Ferrara et al. 1997).

Terrestrial hermit crabs have developed specific abdominal lungs, a vascular network in the thin dorsal integument of the abdomen (Farrelly and Greenaway 2005), while the number of gills and their area decreases with increasing terrestriality in brachyuran crabs (Farrelly and Greenaway 1992, 1993, 1994; Greenaway 1999). The most advanced coconut crab *Birguslatro* has markedly reduced gills, while gas exchange mostly occurs in specific respiration structures (branchiostegal and abdominal lungs) (Greenaway et al. 1988; Farrelly and Greenaway 2005), represented by vascular casts protruding into an aerial chamber, resulting in a large surface area. The diffusion barrier in these structures is shorter and hemolymph from the lungs goes directly to the pericardial sinus; this species also has the highest blood pressure (50 mm Hg) among crustaceans (Greenaway et al. 1988, 1990; Greenaway 2001). The usage of a protective gastropod shell in land hermit crabs favored the evolution of the abdominal lung, while the rejection of this heavy shell by *Birguslatro* also stimulated the development of branchiostegal lungs, which allowed effective colonization of terrestrial and even arboreal habitats (Greenaway 2003; Farrelly and Greenaway 2005). The cuticle of the gill lamellae of almost all air-breathing terrestrial decapods is usually much thicker than that of their aquatic relatives (Taylor and Taylor 1992). The surface of the gills of most terrestrial brachyurans can also be increased by various morphological structures, ranging from the thickening of the marginal canal (*Cardisomahirtipes*), marginal nodular swellings (*Geograpsusgrayi*) (Farrelly and Greenaway 1992), and vascular casts decorating the gill and branchial chamber surface (*Gecarcoideanatalis* and *G.lalandii*) (Cameron 1981; Farrelly and Greenaway 1992; Morris and Dela-Cruz 1998; Morris 2002). Terrestrial grapsid and gecarcinid crabs also have highly developed lung-like structures in addition to their gills, increasing the surface area for gas exchange (Farrelly and Greenaway 1993).

Land crabs and hermit crabs also have physiological changes in the respiratory organs. For example, they have developed a double circulation of hemolymph either through the lungs or through the gills. In addition, one of the functions of the gills in the aquatic environment, namely the exchange of salt and ammonia with water, does not work in terrestrial species, which contributed to the development of other physiological adaptations.

#### Body size

The body size of terrestrial crustaceans does not obey most known biological rules (Karagkouni et al. 2016a, b), except for the above-mentioned relationships with the evaporation intensity. In smaller macrocrustaceans such as landhoppers and woodlice, body size reduction can be considered as an adaptation to living in narrow spaces (Loureiro et al. 2005; Hornung 2011). A positive correlation between body size and latitude is observed in terrestrial crustaceans in arid habitats and partly in a temperate climate (Karagkouni et al. 2016a, b). On the other hand, the terrestrial hermit crab *Birguslatro*, living on tropical Indo-West Pacific oceanic islands with a warm and humid climate, is one of the largest present-day arthropods (Drew et al. 2010, 2013). Tropical South American gecarcinids, *Cardisomaguanhumi*, and *C.crassum*, the largest known forest-dwelling crab species, can reach a maximum width of the carapace of 130 mm and a weight of 500 g (Ehrhardt and Niaussat 1970; Pérez-Chi 2005), while *Tuerkayanahirtipes* from Andaman Islands have a weight of up to 600 g (Alcock 1900; after Green 1997). A wide range of predators apparently preys upon smaller talitroids and woodlice, so that the island environment does not exert strong selection pressure on their body size (Karagkouni et al. 2016a).

It is also worth mention here that an important ecological advantage of crustaceans over other terrestrial arthropods is the growth throughout life and the ability to regenerate limbs, whereas the molting process and subsequent calcification represent an extremely vulnerable stage of their life cycle.

#### Limbs and mobility

Life on land is impossible without the ability to move using principles very different from those used in the water. Most true terrestrial crustaceans are not able to swim as adults and may drown in the water, while they can crawl in a moist environment. Nevertheless, their ability to move over land is determined by the morphology of their aquatic ancestors.

Terrestrial brachiopods (cladocerans) differ from their aquatic relatives by reduced antennae and eyes, stronger armored (spinulated) limbs and the presence of robust teeth on their post-abdominal claw (Cammaerts and Mertens 1999; Chiambeng and Dumont 1999; Sousa et al. 2017).

Terrestrial ostracods also hardly differ in morphology from their aquatic relatives, except for the progressive loss of swimming setae, whereas the second pair of antennae became strong. Together with the fusion of some segments, this makes the limbs better suitable for crawling. This can be considered as a specific adaptation to terrestrial habitats, characteristic of non-related lineages (genera) (De Deckker 1983; Powers and Bliss 1983; Martens et al. 2004; Pinto et al. 2008). All known soil- and leaf litter-dwelling copepods have elongated worm-like bodies, especially typical of canthocamptids that allow them to creep in narrow pores in moist edaphic habitats (Fiers 2013). Diapausing highly protected eggs and cysts produced by Copepoda and Ostracoda allow them to cross wide areas of the land with the help of wind or vertebrates (Vogt 2016; Glime 2017a).

Terrestrial talitroids (Talitroidea) do not move very fast on the ground, but they walk efficiently upright, as well as jump like fleas, which helps to avoid predators (Bracht 1980; Wan and Gorb 2021). The body-catapult mechanism of *Talitrussaltator*, consisting of the arc-shaped structures at the leading edge of the five posterior segments, having fibrous microstructures along it in a circumferential direction and containing a large amount of elastic tissue and a small amount of chitin, can accumulate a large amount of energy to enhance the force of the jump. It has an output power of ~ 1.7–5.7 kW/kg, which is 3.4–11.4 times higher than the limit of the output power of muscles of other arthropods (Wan and Gorb 2021). Woodlice reflexively move faster in dry conditions (unfavorable environments) and slower in wet ones (Fraenkel and Gunn 1961). In addition, woodlice show thigmokinesis, meaning they stop moving when they are close to a solid object, including other individuals, so they often form clusters in humid microsites (Friedlander 1964; Sutton 1972).

Terrestrial crabs are well adapted for fast movement to escape predators, for example, the semi-terrestrial Hawaiian ghost crab *Ocypodeceratophthalmus* is among the fastest known wingless land invertebrates reaching running speed of 3 m sec^-1^ (Burrows and Hoyle 1973; Florey and Hoyle 1976). Unusually long legs of forest crabs of the family Gecarcinucidae allow them to climb trees (Grinang et al. 2015; Biju Kumar et al. 2017; Ng and Ng 2018), and efficiently move along the outcrops of karst massifs (Shih and Do 2014; Do et al. 2016; Huang et al. 2020).

#### Sensory organs (thermal, humidity, olfactory, and visual sensitivity)

Woodlice and talitroids have effective temperature and humidity receptors allowing them to select warm or wet habitats or to avoid extreme temperatures and dry areas (Warburg 1964, 1968, 1993; Morritt 1998; Lagerspetz and Vainio 2007). Some woodlice have specific temperature-sensitive neurons that respond to evaporative heat loss and humidity (Schmalfuss 1998; Hornung 2011). The sensitivity of antennae to temperature may depend on local water loss from thin-walled structures, which probably contain mechanosensory neurons (Sutton 1972, 1980; Hallberg and Skog 2011; Schmidt and Mellon 2011). It is assumed that the antennae, as well as the tritocerebral processing structures associated with them, partially compensate for the loss or significant reduction of the (deutocerebral) primary olfactory pathway in terrestrial talitroids and oniscids (Krieger et al. 2021).

Although the morphological structure of antennae in coenobitid hermit crabs is completely similar to the structure of the chemoreceptive organs of related aquatic species (Ghiradella et al. 1968a, 1968b), their olfactory lobes in the brain are significantly increased compared to their aquatic relatives (Krång et al. 2012; Polanska et al. 2020; Krieger et al. 2021). *Coenobitacompressus* can detect odors of feces, fruit, and fish from a distance of at least 5 m by detecting volatile chemical signals and can detect non-volatile compounds using contact chemoreception (Rittschof and Sutherland 1986; Dunham and Gilchrist 1988). Acetoin from coconut and arenga fruit was the only one of the 15 volatile compounds tested that attracted omnivorous robber crab *Birguslatro* (Knaden et al. 2019), which has an olfactory system similar to that of insects (Stensmyr et al. 2005). In contrast, terrestrial talitroids, woodlice, and crabs have reduced and miniaturized antennae and olfactory aesthetascs, as well as primary olfactory processing brain centers, suggesting a loss of olfaction during the evolution on land (Kuenen and Nooteboom 1963; Krieger et al. 2015, 2021).

Terrestrial crabs and hermit crabs typically have well-developed visual neuropiles and neuronal substrates for a sophisticated analysis of the compound eye input. Vision plays an important role in their behavior such as food and habitat search, mating, and orientation (Krieger et al. 2015; Chou et al. 2020). The fiddler crabs of the genus *Uca* can distinguish colors (Detto 2007; Jordão et al. 2007) and possesses ultraviolet and polarization vision (Detto and Backwell 2009; How et al. 2012), which is an important factor for their orientation and social interactions (Detto et al. 2006; Detto 2007). The visual system of *Coenobita* is separated into peripheral and central viewing areas (Ping et al. 2015).

### ﻿Physiological adaptations

#### Feeding and digestion

Crustaceans are primarily detritophages or herbivores, with the feeding objects ranging from small organic particles extracted from soil to leaf litter, seeds, flowers, and fruits. The problems associated with a low-quality plant diet can potentially be avoided by selecting the most palatable food items. To improve energy efficiency, crustaceans can supplement a plant-based diet with animal tissues, but only a few species became carnivorous. Other trophic strategies of crustaceans include low intake rates, longer retention of digesta, and efficient assimilation of structural carbohydrates (Linton and Greenaway 2007). Many species, especially small-sized macrocrustaceans, are omnivorous and respond to spatial and temporal changes in the quality or quantity of food resources by changing their activity and feeding tactics, including coprophagy (Hassall and Rushton 1982). Effective mechanical crushing of coarse plant material is necessary to increase the surface of the food substrate exposed to digestive enzymes (Johnston et al. 2005). All crustaceans have powerful jaws for the initial crushing of plant material; after swallowing the pieces are further crushed by a gastric mill (Linton and Greenaway 2004). Active cellulases and laminarinases have been identified in the digestive juice, gut, and midgut gland or hepatopancreas of a wide variety of crustacean species allowing them to hydrolyze cellulose and hemicellulose to their constituent sugars (reviewed in Linton and Greenaway 2007). Lichenase and xylanase have been less studied but are present in the digestive juice and may be common in Crustacea (Linton and Greenaway 2004). Isopods depend on the hydrolases of microorganisms (Kozlovskaya and Striganova 1977), including fungal enzymes such as xylanase and cellulase (Kukor and Martin 1986). The dependence of woodlice on the microbial activity of leaf litter, measured in terms of cellulase activity, has been repeatedly demonstrated (Uesbeck and Topp 1995; Zimmer and Topp 1997).

#### Water balance and osmoregulation

The tolerance to desiccation is not likely to be the most important factor contributing to the success of macrocrustaceans in terrestrial habitats (Lazo-Wasem 1984; Moore and Francis 1985). The absence of any specific physiological adaptation in osmoregulation suggests that they were not subjected to the great desiccation stress during the evolution, since otherwise one would expect some capacity to deal with hyperosmotic stresses caused by desiccation (Morritt 1988). The osmotic pressure of the hemolymph and exosomatic fluid (400–850 mOs, usually 700–850 mOs) in most terrestrial species are slightly lower than in seawater, although in talitroid *Makawehurleyi* the mean osmotic pressure of hemolymph is ~ 45% of seawater (Duncan 1985). It was suggested that the lack of ions, especially chloride (Cl-) and sodium (Na+), might restrict the distribution of talitroid species to coastal areas (Spicer et al. 1987; Morritt and Spicer 1998; Richardson et al. 2001a, b, 2003; Cowling et al. 2003, 2004). Water loss due to evaporation over 25% is fatal for beach flea *Orchestiagammarellus* (Moore and Francis 1986b; Morritt 1987), and the threshold of 30% was reported for another species, *Platorchestiaplatensis* (Garces 1988). However, physiological adaptations related to ion regulation were seemingly more important in the evolution of landhoppers than adaptations to resist the effects of desiccation (Friend and Richardson 1986; Moore and Francis 1986b). Some oniscoids and talitroids can absorb water not through the oral route, but through the cuticle (Hoese 1981, 1982; Moore and Richardson 1992).

Almost all land crabs are restricted to tropical and subtropical humid ecosystems, although they depend not so much on the temperature and humidity of the environment, as on these parameters inside the branchial chamber (Wolcott 1988). Fluctuations in ambient temperature and environmental conditions generally favor evaporative water loss, due to increased metabolism (Weinstein 1998). The semi-terrestrial potamonid crab *Sudanonautesafricanus* from wet rain forests of West Africa tolerates water loss of ≤ 20% of body weight (34% of total body water) (Lutz 1969) and coastal hermit crab *Coenobitabrevimanus* up to 28% of total body water (Burggren and McMahon 1981). The Australian desert crab *Austrothelpusatransversa* can lose up to 42% of body weight, having one of the highest rates of water content in tissues among land crabs. Despite these features, the crab can only survive ~ 90 h at a relative humidity of 70% (rH) and 20 °C (MacMillen and Greenaway 1978; Greenaway and MacMillen 1978; Burggren and McMahon 1981). Of the other land crabs studied, only *Gecarcinuslateralis* can tolerate a similar to *Austrothelpusatransversa* degree of weight loss (< 40%) (Bliss et al. 1966). In land crabs and *Birguslatro*, dehydration and changes in hemolymph concentration are resisted using combinations of both behavioral (immersion, burrowing, water storage in the body or branchial chambers, and drinking) and morphophysiological (evolutionary reduction in gill size, urine reprocessing, excretion of nitrogenous waste as urea or uric acid) adaptations (Greenaway 1988; Greenaway et al. 1988; Wolcott 1991).

#### Excretion

The majority of terrestrial crustaceans, like their aquatic ancestors, are ammonotelic, excreting ammonia as the main waste product (Dresel and Moyle 1950; Linton et al. 2017). Only one species, *Birguslatro* is known to be primarily purinotelic, producing white fecal pellets of guanine and uric acid (Greenaway and Morris 1989; Linton et al. 2005, 2017). Even completely air-breathing gecarcinid crabs with well-developed lung-like structures, still require periodic immersion in water to facilitate nitrogen excretion (Adamczewska and Morris 1996; Dela-Cruz and Morris 1997a, 1997b). Ammonia is eliminated either in solution (excretory fluid) or as a gas in woodlice and talitroids (O’Donnell and Wright 1995; Linton et al. 2017). Waste nitrogen is stored as transaminated amino acids such as glutamate, glutamine, and glycine, between excretory bouts (Linton et al. 2017). Terrestrial isopods, amphipods, and decapods have solid purine urate deposits synthesized from excess dietary nitrogen, which are stored inside the connective tissue (Linton and Greenaway 1997, 1998; Linton et al. 2017). It has been suggested that these deposits function as either excreta or temporary nitrogen storage and are generally not used during times of negative nitrogen balance or in situations of high nitrogen demand (oogenesis and molt) (Linton and Greenaway 1997). In woodlice, urates seem to function as a cation store during dehydration or as an antioxidant to prevent oxidative tissue damage (Linton et al. 2017).

#### Physiology of breeding

For most terrestrial macrocrustaceans, reproductive biology and reproduction cycles are generally similar to their aquatic relatives, although a decrease in the number of eggs in parallel with an increase in the egg size (sometimes only one large egg) and reduced (abbreviated) development occurs in some species (Williamson 1951; Steele and Steele 1975; Wildish 1979; Cardoso et al. 2001). Terrestrial crustaceans are mostly iteroparous, while some woodlice are known to be semelparous (Warburg et al. 1993; Linsenmair 2008). All terrestrial macrocrustaceans have internal fertilization, effectively conserving sperm in the female genital tract (e.g., Longo et al. 2011). Talitroids and woodlice have direct development inside an external pouch formed by the brood plates (oostegites) (Richardson et al 2001a, b), without an aquatic larval stage, which is a crucial adaptation for a fully terrestrial lifestyle. The larval development of decapods depends on their origin. Taxa derived from the marine environment still have to release larvae into the sea, returning to the mainland as the last zoeal/megalopa stages, while taxa of freshwater origin mostly have reduced or abbreviated development and can live in terrestrial habitats far from the sea coastline (Bliss 1979; Wolcott 1988). Most terrestrial ostracods are described from asexual populations (Pinto et al. 2005a, 2005b), especially in Darwinulidae, whose lineage was asexual for at least 200 Mya (Martens et al. 2003; Pinto et al. 2004, 2005a, 2005b). Parthenogenesis seems to be a favorable pre-adaptation, since most known terrestrial ostracods exhibit very low densities (with some exceptions, such as Brazilian *Penthesilenula*) and are unable to move over long distances (Pinto et al. 2004, 2005b). Terrestriality of some ostracods may involve the protection of fertilized eggs from desiccation. Developing embryos of the moss-dweller *Scottiaaudax* can be preserved in the maternal shell until they become free-living juveniles (Chapman 1961; Glime 2017a).

#### Low metabolism and longevity

Slow growth and longevity increase the time available for the accumulation of dietary nitrogen and other nutrients required for the growth of animals. For example, the minimum intermolt nitrogen requirement of *Gecarcoideanatalis* is only 4.8±1.7 mmol N/kg dry body weigh/day due to a low rate of basal protein catabolism (0.12±0.04% total body protein/day) and low fecal nitrogen concentration (38–56 mmol N/kg of dry weight) (Linton and Greenaway 2000). This way, *G.natalis* can cover the nitrogen requirements of intermolt, molt, and oogenesis from its nitrogen-poor leaf litter diet (Linton and Greenaway 2000, 2007). Mass-specific metabolic rates of animals and thus basal protein catabolism and minimum nitrogen requirements decrease with increasing body size. Life spans for many terrestrial herbivorous crabs are long with estimates of 20+ years for *G.natalis* (Green 2004) and *Cardisomaguanhumi* (Wolcott 1988), 12+ years for *Coenobitaclypeatus* (Chace 1972), and 8–17 years for *Ucidescordatus* (Pinheiro et al. 2005). Longevity is seemingly linked to large body size in the gecarcinids. The lifespan of *Birguslatro* is estimated as 40–60 years (Greenaway 2003). Sexual maturity in large land crabs is not attained until 3–4 years of age (Henning 1975; Wolcott 1988; Green 2004; Pinheiro et al. 2005).

#### Other physiological adaptations

Terrestrial microcrustaceans are fragmentarily studied for any physiological adaptations, although the absence of hemoglobin is considered an adaptation for a bryophytic lifestyle in harpacticoids. This suggests that oxygen is present in sufficient amounts and energy-requiring development of the pigment is not necessary (Green 1959; Glime 2017a, b). Land crab *Ocypodequadrata* can maintain its body temperature lower than the ambient air temperature using its enlarged claws and evaporation from the surface of the exoskeleton (Weinstein and Full 1994; Weinstein et al. 1994). Semi-terrestrial crabs of the genus *Uca* also use their enlarged claw for heat transfer to or from the environment (Windsor et al. 2005). Some woodlice evolved specific chemical (gland secretions and accumulation of potentially toxic concentrations of metals in their body tissues), morphological (heavily incrusted armor), and behavioral defenses (rolling into a ball or clinging to the substrate) as protection from specialized predators (Sutton 1972; Deslippe et al. 1996; Schmalfuss 1984).

### ﻿Specific behavioral adaptations

#### Regulation of temperature and humidity

Shore woodlouse *Ligiaitalica*, living on and under rocks along the Mediterranean coasts, is strongly photonegative at temperatures of 20–30 °C, somewhat less at 6–10 °C, and photopositive at 40 °C when forced to leave rock crevices to find a cooler environment (Perttunen 1961). Desert-dwelling woodlice can maintain their heat and water exchange within their physiological tolerance limits by nocturnal activity and the ability to roll up into an almost perfect sphere thus preventing moisture loss (Linsenmair 1985, 1987, 2008; Shachak 1980; Shachak and Newton 1985). Conglobation is considered a mechanism preventing evaporation since the water loss rate is decreased significantly (up to 35%) by this behavior, depending on relative humidity (Smigel and Gibbs 2008).

Gecarcinidae land crabs are diurnal and nocturnal, but their activity is always positively correlated with relative humidity (Green 1997), and increases during the wet season, when humidity, and also the availability of seeds and seedlings are higher (Capistrán-Barradas et al. 2003; Sherman 2003; Lindquist and Carroll 2004). With the risk of desiccation, the activity of land crabs decreases when the humidity falls below 88% and stops below 77% (Green 1997; Hicks 1985). The surface soil temperatures (compared to air temperature) have a significant negative impact on the crab activity and abundance (Govender et al. 2008). Many land crabs spend the daytime inside their burrows, avoiding high surface temperatures (Atkinson and Taylor 1988). For example, at an air temperature of 35 °C and surface soil temperature of ~ 48–50 °C, the temperature inside burrows of land crab *Gecarcinuslateralis* at 40 cm depth ranged within 28–32 °C, providing sufficient protection from high temperatures and low humidity (Bliss 1968; McMahon and Burggren 1988; Govender et al. 2008). The burrowing in the humid soil allows some species to survive with little or no access to freestanding water (Greenaway and MacMillen 1978; Greenaway 1994). However, high ground water levels often preclude deep burrowing, which reduces the habitat of some forest crab species (Govender et al. 2008). Aestivation (summer sleep) is known for desert woodlice (Edney 1964) and terrestrial crabs (MacMillen and Greenaway 1978; Storey and Storey 2012). Desert-dwelling crabs able to aestivate can remain inside their clay-plugged burrows for up to 6 years, waiting for the rain (Ng et al. 2008).

Some desert woodlice have developed social behavior, diurnal activity, and semelparous reproductive strategy (Linsenmair 1974, 1985, 1987, 2008; Caubet et al. 2008; Hornung 2011). The advantage of semelparous reproduction in this case is apparently to invest all the accumulated resources in one reproductive effort since the chances of finding suitable conditions in the deserts are small. Aggregation is also considered one of the adaptive mechanisms against desiccation (Devigne et al. 2011; Broly et al. 2012, 2013b, 2014) and may create a local humid microclimate for all individuals in a small volume (Schliebe 1988). Additionally, aggregation stimulates reproduction in females, accelerating their vitellogenesis (Caubet et al. 1998) and growth (Takeda 1980), which is probably controlled by specific pheromones (Kuenen and Nooteboom 1963; Takeda 1980).

#### Feeding activity

Saprophagous or herbivorous crustaceans tend to select food items of higher quality that contain substantial amounts of easily digestible lipids, carbohydrates, and proteins. For instance, *B.latro* consumes mainly fruits, seeds, and animal material, and practices a highly selective feeding strategy using sophisticated olfactory sense (Fletcher et al. 1990; Hicks et al. 1990; Greenaway 2001). The South African woodlouse *Alloniscusmarinus* exhibits unusual arboreal feeding behavior by eating the green leaves of the bietou bush *Chrysanthemoidesmonilifera*, but not the forest litter (Glazier and Kleynhans 2015). Some crabs feed by rasping leaf tissue from the upper or lower surface of the leaves (Cannicci et al. 1996; Erickson et al. 2003). This can enhance the quality of ingested material by increasing the ratio of mesophyll tissue to indigestible lignin and cutin compared with ingestion of whole leaf material. Intraspecific competition between *Cardisomaguanhumi* in Florida is such that crabs rush from their burrows to compete for falling leaves (Herreid 1963). Gecarcinid crabs also store leaves in their burrows where fungi and bacteria rapidly colonize them, but there is no quantitative data on the rate of utilization of the processed litter (Herreid 1963; O’Dowd and Lake 1989; Green et al. 1999). Predation and cannibalism in *Gecarcinuslateralis* increase when animals are maintained on a low-nitrogen diet (Wolcott and Wolcott 1984, 1987, 1988). Ambush predation is known for terrestrial crayfish (Graham et al. 2022). Such feeding behavioral adaptations are likely to be widespread in terrestrial crustaceans but remain poorly studied.

#### Parental care

Females of some woodlice provide maternal care to eggs and young, supplying nutrients and providing mancae (early-instar juveniles) with an aqueous environment in the modified marsupium (Warburg 1987; Warburg and Rosenberg 1996), which is unique among terrestrial arthropods (Surbida and Wright 2001; Kight and Nevo 2004; Lardies et al. 2004). Marsupium of some terrestrial woodlice contains lipid globules in cotyledons that secrete the marsupial fluid and supply juveniles with nutrients (Hoese and Janssen 1989; Csonka et al. 2015). Maternal care in talitroids includes controlling the osmotic environment of the pouch, cleaning eggs, and perhaps feeding young in a brood pouch (Morritt and Richardson 1998; Richardson et al 2001a, b). Jamaican snail crab *Sesarmajarvisi* breeds inside water-filled shells of the land snail of the genus *Pleurodonte* and provides parental care for larvae (Diesel and Horst 1995; Diesel and Schubart 2000). Vampire crab *Geosesarmanotophorum* and some other species of the genus from high-altitude forests of Sumatra exhibit a completely abbreviated development and unusual brooding behavior in which the female carries her offspring on the dorsal surface of the carapace for approximately 2–3 days after hatching (Ng and Tan 1995; Ng et al. 2015b; Ng and Ng 2019). The most interesting case of parental care is described in the Jamaican crab *Metopauliasdepressus*, which includes the long-term maintenance of optimal levels of appropriate conditions (oxygen, pH, and calcium (Ca+)) for larval development (Diesel 1989; Diesel and Schuh 1993). These eusocial crabs live in large colonies consisting of the mother and her offspring, where the older offspring participate in the colony defense, and young adult females remain in their natal colony as subordinate (non-reproductive) females, with the prospect of inheriting their mother bromeliad as a breeding site (Diesel and Schubart 2000, 2007; Vogt 2012).

#### Breeding migrations

One of the most important features and adaptations of land gecarcinid crabs is the annual migration to the coast to release their eggs into the ocean (Adamczewska and Morris 1996, 2001; Morris 2005; López-Victoria and Werding 2008), with the most exciting migrations of breeding females known for *Gecarcinusruricola* in Providence Island (Hartnoll and Clark 2006), *Gecarcoideanatalis* in Christmas Island (Hicks 1985; Hicks et al. 1990) and *Gecarcinuslateralis* in Florida and Bermuda (Bliss et al. 1978). During these migrations, crabs can travel up to 5 km daily for many days to reach the coastline and must maintain moving for extended periods, up to 12 h each day (Hicks 1985; Adamczewska 1997; Green 1997). In land crabs from arid or semi-arid habitats, young individuals grow very quickly after hatching, and then return to the ground and build their burrows at the beginning of the dry season (McCann 1938). Very high fertility in land crabs, compared to any of soil inhabitants, for example, 19,000–109,000 eggs in *Gecarcinuslateralis*, determines the high reproductive potential that ensures the prosperity of crabs in terrestrial habitats (Green et al. 1997).

Behavioral adaptations are likely to predominate over morphological and physiological ones, including, for example, avoiding harsh conditions, social and specific breeding behavior with migrations to water, and other behavioral patterns. The main strategies in the hot and dry climate include minimizing water evaporation by seeking shelter and having a nocturnal lifestyle. At the same time, most of the morphological, physiological, and behavioral adaptations presented above suggest that terrestrial crustaceans are evolving and adapting to terrestrial habitats, but still have a range of strong limitations hampering their wider distribution and dominance in terrestrial ecosystems.

#### Additional literature on the topic

Most studied are adaptations of woodlice for their terrestrial lifestyle such as reduction in size (Hornung 2011), specific sensitive structures (Hornung 2011), cuticle structure (Bursell 1955; Schmalfuss 1978; Holdich 1984), surface structures (Holdich and Lincoln 1974; Holdich 1984), pleodopodal lungs (Cloudsley-Thompson 1988; Schmidt and Wägele 2001; Paoli et al 2002; Wright and Ting 2006) and brood pouch structure (Hoese 1984). Numerous reviews treat various aspects of arthropod terrestrialization, including locomotion (Weihmann 2020) and other behavioral adaptations (Warburg 1968; Powers and Bliss 1983; Lagerspetz and Vainio 2007), chemoreception and thermoreception (Ache 1982), general physiology (Carefoot 1993; Greenaway 1999), the evolution of the olfactory system (Krieger et al. 2015, 2021), respiration (McMahon and Burggren 1988; Morris 2002), nitrogenous waste metabolism (Morris 2002; Linton et al 2017), water and hemolymph conducting system (Horiguchi et al. 2007), nutrition (Zimmer and Topp 1998b; Zimmer 2002a), diseases (Provenzano 1983; Federici 1984), and specific adaptation to the arid environment (Cloudsley-Thompson 1975).

General review of adaptations of Crustacea to land are presented in Edney (1968), Bousfield (1968), Bliss and Mantel (1968), Cloudsley-Thompson (1988), Morritt and Spicer 1998; Dunlop et al. (2013), Richardson and Araujo (2015), Glime (2017a,b), and Sfenthourakis et al. (2020).

### ﻿Trophic interactions and role in the ecosystems

The feeding activity of macroinvertebrates is considered one of the most important initial processes of the decomposition of organic matter (Ott et al. 2012; Griffiths et al. 2021; Potapov et al. 2022). Terrestrial macrocrustaceans, along with other macroarthropods (millipedes, termites) and earthworms, can be cumulatively classified as primary decomposers and as ecosystem engineers that substantially modify the physical structure of plant litter and soil (Jones et al. 1994; Lawton and Jones 1995; Lavelle et al. 1997).

#### Litter consumption and decomposition

Terrestrial decapods living in coastal forests forage primarily on plant material such as leaf litter (Kellman and Delfosse 1993; Sherman 2003), fruits (Capistrán-Barradas and Moreno-Casasola 2006), seeds (Garcia-Franco et al. 1991; Lindquist and Carroll 2004) and seedlings (Green et al. 1997; Sherman 2002). The huge densities of land decapods (crabs and hermit crabs) on oceanic islands lead to the removal of a significant amount of litterfall and changes in the structure of the nutrient cycles (O’Dowd and Lake 1989; Kellman and Delfosse 1993). Decapods sometimes monopolize litter recycling (Green et al. 1997). For instance, the Bermuda blackback land crab *Gecarcinuslateralis* may consume 75–97% of the leaf litter biomass available for decomposition (Kellman and Delfosse 1993); the land crab *Gecarcoideanatalis* processes 39–87% of annual litterfall on Christmas Island (Green et al. 1999).

Smaller terrestrial macrocrustaceans, such as talitroids (Friend 1975; O’Hanlon and Bolger 1999; Handa et al. 2014) and woodlice (Hassall and Sutton 1978; Hassall et al. 1987; Mocquard et al. 1988; Špaldoňová and Frouz 2014) are also highly efficient detritivores. They can consume 6–55% of the total litterfall, playing an important role in litter decomposition and nutrient mineralization. Fully terrestrial woodlice may compete with other saprophagous soil animals for high-quality food resources (Rushton and Hassall 1983), whereas competition between sympatric species may be reduced by species-specific nutritional requirements and digestive capabilities (Zimmer et al. 2002).

Crustaceans enhance litter decomposition both directly, via consumption and assimilation and indirectly by fragmenting and increasing the surface area available for colonization by saprotrophic microbiota and stimulating microbial activity in their feces (Coughtrey et al. 1980; Teuben and Roelofsma 1990). As could be expected, woodlice are best studied in this respect. Leaf litter eaten and digested by isopods differs physically and chemically from intact leaves (Hassall et al. 1987; Gunnarsson et al. 1988). Increased microbial activity in the gut and fresh feces (Zimmer 2002a, 2002b; Zimmer et al. 2002, 2003) ensures the degradation of cellulose (Hartenstein 1964; Zimmer and Topp 1998b; Zimmer and Brune 2005) and even phenolic leaf litter compounds (Neuhauser and Hartenstein 1976; Zimmer and Topp 1998a; Zimmer 1999). Passing through the digestive canal, saprotrophic microflora changes both in density and in species composition (Hartenstein 1964, 1982; Coughtrey et al. 1980; Ineson and Anderson 1985). Recent studies of gut microbiota in the woodlice *Armadillidiumvulgare* and *Porcellionidespruinosus* revealed a very diverse bacterial community that varies between host populations, suggesting an important proportion of environmental microbes in the gut-associated microbiota (Bouchon et al. 2016; Delhoumi et al. 2020). Microbial inoculation of leaf litter increased litter palatability (Hartenstein 1964; Hassall et al. 1987; Rushton and Hassall 1983) and quality (Ullrich et al. 1991; Uesbeck and Topp 1995; Zimmer and Topp 1997, 2000) by reducing the C/N ratio and/or quantity of phenolic compounds (Kuiters and Sarink 1986; Poinsot-Balaguer et al. 1993; Zimmer 1999, 2002a). Noteworthy, woodlice cannot separate low- and high-quality litter (i.e., oak vs. alder) immediately after leaves had been shed, but can do so after early stages of microbial decomposition, since the microbiota or their waste seem to indicate high-quality food (Zimmer et al. 2003).

The consumption and bioturbation of leaf litter affect the chemical composition and rate of oxygen saturation of the soil, accelerate the decomposition processes, and stimulate the activity of fungi and bacteria (Richardson and Morton 1986; Griffiths et al. 1989; van Wensem 1989; Teuben and Roelofsma 1990; Kautz et al. 2000). Talitroids can reduce the rate of leaching of cations, possibly because cations are bound in the compact fecal pellets produced by landhoppers (Richardson and Morton 1986). An increase in ATP turnover in the *Spartina* litter grazed by the landhopper *Orchestiagrillus* leads to an increase in nitrogen reserves, which is important for the long-term health of the coastal forests (Lopez et al. 1977).

#### Bioturbation

The burrowing activity of terrestrial crustaceans is one of their main ecological functions. Smaller species have less of an effect, although the burrowing activity of woodlice of the genus *Hemilepistus* appears to be an important factor in soil formation in arid regions (Kozlovskaya and Striganova 1977). Forest crabs and crayfish can significantly affect forest ecosystems by increasing soil aeration through burrow construction (Richardson 1983; Green 2004; Pérez-Chi 2005; Gutiérrez et al. 2006), as well as removing the leaf litter and causing local nutrient enrichment of the soil by gathering leaves around or inside their burrows (O’Dowd and Lake 1989; Sherman 2003, 2006). Crayfish *Creaserinusgordoni* annually moved over 80 metric tons of soil ha/yr and created 29–49 km/ha of subterranean tunnels (Welch et al. 2008). Burrows of *Procambarushagenianus* may extend 4 m below the surface (Fitzpatrick 1975), and in suitable habitats, there may be at least one crayfish burrow system per square meter (Reynolds et al. 2013). The activity of the East African land crabs *Neosarmantiummeinerti* and *Cardisomacarnifex* is limited to the upper 20 cm of soil, where it affects ~ 0.07% of the soil daily or about 25% per year (Micheli et al. 1991). In another study, combining estimates of burrow volume, density, and turnover suggest that red crabs *Gecarcoideanatalis* can increase the surface area of soil available for gas exchange by ~ 13%, although for one year they probably turn over < 1% of the top 20 cm of the forest soil in Murray Hill, Christmas Island (Green 1997). The physical removal of litter from the surface to deeper and moister soil layers may be one of the most important indirect contributions to decomposition processes (Hassall et al. 1987). The litter mass was 5.0–5.6 times higher in crab exclosures than in control open plots (Kellman and Delfosse 1993; Sherman 2003). Leaf litter caching inside the burrows is common in gecarcinid land crabs (Fimpel 1975; Henning 1975; O’Dowd and Lake 1989), but the accumulation of leaf litter around the entrance is described only in *Gecarcoideanatalis* (O’Dowd and Lake 1989; Green 2004). Burrows of large land crabs such as *Cardisomaguanhumi* are durable and turn over very slowly (Herreid and Gifford 1963; Green 2004). Fairly long-lived burrows, with an average turnover time of more than 4 years create a mosaic of nutrient hotspots potentially useful for seedling growth (O’Dowd and Lake 1989; Green 2004). They can also have a significant effect on carbon sequestration, and since the soil is enriched with nutrients, the density of plant roots is higher around burrowing microsites in mainland forests (Sherman 2006). Other burrowing terrestrial crustaceans, such as crayfish, perform similar ecological functions (Kingwill 2008; Loughman 2010; Bryant et al. 2012). These data suggest that forest crabs and crayfish may have a somewhat different effect on aboveground processes compared to crabs in tidal habitats, which constantly dig numerous small burrows (Bertness and Miller 1984; Smith et al. 1991), thus constantly aerating the substrate, and sometimes even draining swampy hypoxic soils (Katz 1980; Montague 1980, 1982; Takeda and Kurihara 1987).

#### Plant recruitment

Woodlice *Armadillidiumvulgare* and some other species are partly granivorous, in some habitats being efficient predators of seed of *Taraxacum*, *Capsella*, *Poa*, and other plants (Saska 2008; Honek et al. 2009; Singer et al. 2012). *Australiodillobifrons* and *Porcellioscaber* feed on wheat seedlings under laboratory conditions and probably can cause significant damage to wheat seedlings when reaching very high densities in the field (Paoletti et al. 2008). The activity of land hermit crabs and forest crabs may be a major factor controlling plant communities through feeding on seeds and seedlings, recycling nutrients, and affecting tree density and size structure (Louda and Zedler 1985; O’Dowd and Lake 1991; Green et al. 1999; Lindquist et al. 2009). Land crabs greatly affect seedling recruitment in semi-deciduous seasonal dry tropical forests (Delfosse 1990; Kellman and Delfosse 1993). For example, land crabs *Gecarcoideanatalis* grazed 25 seedling species on Christmas Island, processing more than 80% and eating ~ 47% of them (Green et al. 1997). Seedling density was 20-fold higher, and seedling richness was 5-fold higher in crab eхclosures than in unfenced control plots (O’Dowd and Lake 1990; Green et al. 1997). In mainland tropical forests, seedling density increased by 144% in crab exclosures (Sherman 2002). Annual fluctuations in the density of the crab population may allow pulses of tree recruitment in “low crab” years (Green et al. 1997, 2008; Sherman 2002; Lindquist and Carroll 2004). Indirectly, the removal of leaf litter by crabs can change the visibility of seeds to predators, as well as the micro-environmental conditions for seed germination and seedling establishment (Kellman and Delfosse 1993). Leaf litter depth and tree seedling density are negatively correlated with the burrow density of land crabs *Gecarcinusquadratus* in Costa Rica (Griffiths et al. 2007). Land crabs differentially prey on seeds and seedlings along nutrient, chemical, and physical environmental gradients, and crab consumption has primacy over many environmental factors, acting as the main limiting factor of tropical tree recruitment, and affecting the structural and compositional organization of coastal forests (Green et al. 1997).

#### Predators

Terrestrial microcrustaceans are involved in complex trophic relationships, although to date they have been studied fragmentary. The cosmopolitan soil harpacticoids *Phyllognathopusviguieri* actively prey on different species of soil nematodes using their modified leaf-like maxillipeds (Lehman and Reid 1993). Adults and copepodites of *Virbiocyclopssilvaticus* occasionally consumed nematodes and injured oligochaetes (Rocha and Bjornberg 1988). Soil harpacticoid *Epactophanes* sp. are classified among bacterial-feeding organisms but may also feed on nematodes (Birch and Clark 1953; Reid 2001).

Woodlice can prey on smaller animals, e.g., insect larvae. For example, *Armadillidiumvulgare* were observed feeding on pupae of fruit flies *Drosophilamelanogaster* in citrus orchards in California, although alternative food was abundant (Edney et al. 1974). Large coconut crab *B.latro* can prey on other land crabs (Krieger et al. 2016), birds, and rats (Kessler 2005; Laidre 2017). The land crab *Gecarcinuslateralis* is a significant predator of the abundant Bahamian land snails of the genus *Cerion* (Quensen III and Woodruff 1997), crab *Rodriguezusgarmani* was observed to consume snakes (Maitland 2003), large land crabs are major predators of nesting sea birds (Paulay and Starmer 2011). Land hermit crabs (*Coenobita* spp.) and large gecarcinid crabs have been reported to feed on an extremely wide dietary spectrum, including dead animals washed into the tidal zone and their feces (Burggren and McMahon 1988; Dunham and Gilchrist 1988; Thacker 1996). High abundance, along with the ability to dispose of all organic residues on the coast and in the surrounding forest in a very short time (Hsu et al. 2018) suggests the importance of crabs as consumers of carrions (Degener and Gillaspy 1955; Niering 1956, 1963; Wiens 1962; Page and Willason 1983). For instance, *Coenobita* spp. potentially control fly populations by rapid removal of carrions. In areas where hermit crabs were common, the flies were seemingly less numerous than in areas where hermit crabs were absent (Page and Willason 1982, 1983). Small *Geosesarmamalayanum* and *Geosesarmaperaccae* crabs climb into the pitchers of *Nepenthesampullaria* and eat the prey, but sometimes they fall into the trap and die (Ng and Lim 1987; Ng 1988).

#### Prey

Knowledge of the position of microcrustaceans in terrestrial food webs is extremely limited. In wet habitats, they are likely consumed by predators along with other microarthropods. For example, terrestrial harpacticoids are among the main prey items of arboreal wandering salamanders *Aneidesvagrans* living in wet bryophytes more than 80 meters above the forest floor in the Californian redwood forest in the USA (Camann et al. 2011). Larger crustaceans are readily consumed by a wide array of vertebrate and invertebrate predators. Generalist predators rarely feed on woodlice (Gorvett 1956), but such cases are known for hedgehogs (Shilova-Krassova 1952), shrews (Perneta 1976), moles (Godfrey and Crowcroft 1960), frogs, toads, lizards, birds, and some predatory arthropods (Sunderland and Sutton 1980; Bureš and Weidinger 2003). On the other hand, ants of the tropical genus *Leptogenys* (Dejean 1997; Dejean and Evraerts 1997), spiders of the Palaearctic genus *Dysdera* (Pollard et al. 1995; Rezáč and Pekár 2007; Pekár and Toft 2015) and toad bugs *Nerthramacrothorax* possess adaptation for the effective capture of armored woodlice and detoxication mechanisms alleviating feeding on woodlice (Sunderland and Sutton 1980; Pekár et al. 2016). Approximately 15 other spider species from ten families are suggested to be specialized woodlice predators (Bristowe 1941, 1958; Uhlenhaut 2001; Rezáč et al. 2008). The desert scorpion *Scorpiomaurus* is the main predator of *Hemilepistusreaumurii*, which may comprise 70% of the scorpion’s diet (Dubinsky et al. 1979; Ward 2009). The nocturnal lifestyle of forest talitroids is sometimes explained by the minimization of dehydration as well as predation by birds (Friend and Richardson 1986).

Raccoons, coatis, mongooses, cats, foxes, herons, and other migrating and local birds feed on land crabs in mainland habitats (Sherman 2002; Lindquist and Carroll 2004). In places where there are no large predatory mammals or birds, land crabs may be released from the predator pressure, although the coconut crab *B.latro* hunts on land crabs on tropical islands (Alexander 1979; Hicks et al. 1990; Pérez-Chi 2005). The invasive yellow crazy ant *Anoplolepisgracilipes* have significantly affected the population of the red land crab *Gecarcoideanatalis* on Christmas Island (O’Dowd et al. 2003; Green et al. 2004; Abbott 2005). This had a cascading effect on native species populations at several trophic levels (O’Dowd et al. 2003). Invasive predatory nemertean *Geonemertespelaensis* significantly declined populations of terrestrial crustaceans on some Japanese islands (Shinobe et al. 2017). The potential for invasion meltdown following the local extinction of crab populations suggests that land crabs are a keystone species in the tropical forests of oceanic islands (O’Dowd et al. 2003).

Coconut crabs and crabs of the genera *Cardisoma* and *Ucides* are a widely recognized food source for humans (Carpenter and Niem 1998; Alves et al. 2005; Firmo et al. 2012; Maynard and Oxenford 2014) and have ethno-medicinal significance (for example, Rana 2018), which is a rare case for saprophagous invertebrates from terrestrial ecosystems.

Antipredatory strategies among terrestrial crustaceans are very diverse and include tonic immobility, aggregation or sticking to the ground, the release of strongly acidic secretions, jumping, and effective escape (see review in Tuf and Ďurajková 2022). In addition, they can team up with other individuals, and use stridulation (Cividini et al 2020). Some species developed prickly tergites and aposematic coloration or posture. Most of these strategies have not yet been studied in detail.

#### Parasites and macrosymbionts

Many internal parasites of terrestrial crustaceans are similar to those of their aquatic relatives. There are however specifically terrestrial parasites, such as ~ 150 species of widely distributed Rhinophoridae flies (Insecta: Diptera) known as specialized parasitoids of woodlice (Pape and Arnaud 2001; Nihei 2016; Wood et al. 2018). Two Caribbean flies, *Drosophilacarcinophila* and *Drosophilaendobranchia*, live exclusively on gecarcinoid land crabs, while the Christmas Island fly, *Lissocephalapoweilli*, lives on both crabs and hermit crabs, including *B.latro*, completing their larval development on or inside their crustacean hosts (Stensmyr et al. 2008). Parasitic relationships between an unidentified species of Sphaeroceridae (Diptera) and the land crab *Cardisomacrassum* are known from Cocos Island, Costa Rica (Gуmez 1977). Specialized Cancrincolidae copepods (Copepoda, Harpacticoida) are associated with large land crabs, living inside their branchial chambers (Huys et al. 2009).

Numerous mermithid nematodes, rotifers, rickettsia and other bacteria, and viruses are known from woodlice and land crabs (Provenzano 1983; Federici 1984; Rigaud and Moreau 2004; Wang 2011; Ugbomeh and Bajor 2015). In particular, common iridoviruses (Iridoviridae) confer an iridescent blue color to the body of the infected woodlice (Williams 2008).

Burrows of land crabs and crayfish provide habitat for obligatory and facultative arthropod symbionts (e.g., Bright and Hogue 1972; Horwitz and Knott 1991), including various flies and mosquitoes (Carson and Wheeler 1973; Carson 1974; Gómez 1977; Bertrand 1979; Goettel et al. 1981). *Gramastacusinsolitus*, a very small non-burrowing Australian freshwater crayfish, survives droughts in the burrows of larger burrowing crayfish *Geocharaxfalcata* and *Cheraxdestructor* (Johnston and Robson 2009). Mosquitoes of the genus *Deinocerites* use the upper portions of burrows of large forest crab *Cardisomaguanhumi* as daytime resting sites, while their larvae develop in the water that accumulates at the bottom (Downes 1966; Adames 1971; Wolcott and Wolcott 1990). In turn, killifish *Rivulusmarmoratus* feed on larvae of *Deinocerites* inside the burrows (Taylor 1990). Several woodlice taxa are associated with the nests of social insects, ants, and termites, showing specific morphological (reduction/absence of eyes and body pigmentation) and behavioral (evasive movements) adaptations tolerated by the hosts (Ferrara et al. 1988; Taiti and Ferrara 1988; Kronauer and Pierce 2011; Parmentier et al. 2017; Taiti 2018). Massasauga rattlesnakes (*Sistruruscatenatus*) commonly choose burrows of cambarid crayfish in southeastern Canada as a hibernation site during the winter (Maple 1968; Seigel 1986; Kingsbury 1996, 1999). Subterranean flowers of *Aspidistraelatior* are allegedly pollinated by collembolans and landhoppers *Platorchestiajaponica* (Kato 1995; Conran and Bradbury 2007), although recent studies suggest that pollination is performed by fungus gnats (i.e., *Cordylasixi* and *Bradysia* sp.) rather than crustaceans (Suetsugu and Sueyoshi 2018).

#### Migrations and energy flows

Among the most prominent and significant ecological functions of terrestrial decapods are migrations, one of the most famous and well-studied examples of the invertebrate-mediated transport of organic matter and nutrients from marine to terrestrial ecosystems and back (Klaassen 1975; Wolcott 1988; Hicks et al. 1990; López-Victoria and Werding 2008; Lindquist et al. 2009). Less studied connections can represent ecologically significant lateral links between terrestrial and freshwater ecosystems. Semi-terrestrial gecarcinucid crabs from Asian streams are saprophages (Ng 1989; Lim 2013; Ng and Yeo 2013) feeding on coarse organic matter from neighboring land, e.g., leaf litter, often being the main macrodecomposer (Hill and O’Keeffe 1992; Abdallah et al. 2004; Dobson et al. 2007). They are also opportunistic predators that feed on smaller aquatic organisms (Abdallah et al. 2004; Dobson 2004), being in turn a food source for larger terrestrial animals (Ng 1989), thus carrying out the energy transfer between river and forest ecosystems.

### ﻿Methods used for sampling of terrestrial crustaceans

Methods of collecting terrestrial macrocrustaceans (mainly woodlice and landhoppers) do not differ significantly from those designed for collecting soil macrofauna, such as Macfadyen extractors (Macfadyen 1961), pitfall trapping, or hand-sorting of soil samples. Other methods that are occasionally used include ‘cryptozoa boards’, i.e., artificial shelters placed on the soil surface (e.g., Hodge and Standen 2006). In contrast, sampling microcrustaceans requires specifically designed approaches. Insufficient knowledge of the diversity and ecology of microcrustaceans is clearly related to the lack of adequate and well-known methods for qualitative and especially quantitative sampling of these animals. Small crustaceans can be extracted by flotation or hand-sorting of alcohol-fixed material under the dissecting microscope (e.g., Bernier and Gillet 2012), but these methods are rarely used. Common “dry” extractors (Berlese or Tullgren funnels) used to collect microarthropods are not suitable because microcrustaceans are essentially aquatic animals. “Wet” extractors (Baermann funnels) are designed mainly for nematodes and enchytraeids having thin and smooth bodies and are likely ineffective for quantitative sampling of soil microcrustaceans.

The qualitative methods for collecting terrestrial crustaceans listed below are borrowed mainly from sampling methodologies targeting meiofauna, underground (subterranean) and hypogean microcrustaceans, and collecting burrowing crustaceans such as crabs and crayfish. Many quantitative methods designed for marine and freshwater benthic animals are probably also applicable to quantitative sampling of terrestrial crustaceans (e.g., Boxshall et al. 2016; Hughes and Ahyong 2016). A detailed account of the common extraction techniques of small crustaceans from the ground is given in Pfannkuche and Thiel (1988) and Boxshall et al. (2016). The technique of the wet sieving adapted for the sampling of soil- and leaf litter-dwelling copepods and other small crustaceans is presented in Kikuchi (1984) and Fiers and Ghenne (2000). Generally, a portion of the soil or leaf litter is placed in a beaker or bucket with water and agitated, and the supernatant is decanted into a stack of sieves, with the coarsest sieve at the top to remove larger pieces of detritus. The target size fraction is retained on the ﬁnest sieve and subsequently inspected using a light microscope. Sampling from waterlogged soils can be carried out by pumping and using a mesh for filtration (Hahn 2002; Leijs et al. 2009; Boxshall et al. 2016).

Research on and sampling of burrowing land crabs and crayfish are hampered by their nocturnal activity and underground lifestyle. Commonly used methods include burrow excavation (Ridge et al. 2008; Loughman 2010), opera house and drop nets (Bryant et al. 2012), pitfall traps (Shaw 1996), the Norrocky traps that capture crayfish at the entrance of the burrows (Norrocky 1984; Welch and Eversole 2006; Ridge et al. 2008), the burrowing crayfish net (Welch and Eversole 2006; Kingwill 2008; Ridge et al. 2008), and some others (see review in Bryant et al. 2012). Sometimes these methods are combined; a relatively recent emerging method involves the use of Alka-Seltzer/Aspirin tablets and soda water poured into the burrows with visible water or trialed in conjunction with flooding of burrows with water (Bryant et al. 2012).

## ﻿Conclusions

Terrestrial crustaceans from six main lineages, representing ~ 4,900 currently known species, are widespread in terrestrial ecosystems. The diversity and ecology of terrestrial crustaceans have been studied to various degrees; in particular, the biology of microcrustaceans is still known fragmentarily. Woodlice, the most successful terrestrial crustaceans, have been able to adapt and colonize a wide range of diverse terrestrial habitats, including extreme ones. An array of morphological and physiological limitations (e.g., the absence of a waxy cuticle protecting against evaporation, and aquatic mode of reproduction), likely prevent most other lineages of crustaceans from competing with other terrestrial arthropods and achieving a wider distribution. Due to the high abundance and density in some terrestrial habitats, such as temperate and especially tropical coastal forests and islands, crustaceans often play important ecological roles, being ecosystem engineers and crucial components of food webs, including the upper trophic levels. In many other ecosystems, the diversity and ecological significance of terrestrial crustaceans, especially microcrustaceans, can be significantly underestimated. Although often neglected by soil ecologists, a full diversity of terrestrial crustaceans, besides isopods, should be regarded as a prominent component of soil communities.

## ﻿Additional information

### Author ORCIDs

Ivan N. Marin https://orcid.org/0000-0003-0552-8456

Alexei V. Tiunov https://orcid.org/0000-0003-2400-3559

### Data availability

All of the data that support the findings of this study are available in the main text.
